# Assessment of burnout in veterinary medical students using the Maslach Burnout Inventory-Educational Survey: a survey during two semesters

**DOI:** 10.1186/s12909-014-0255-4

**Published:** 2014-11-28

**Authors:** Munashe Chigerwe, Karen A Boudreaux, Jan E Ilkiw

**Affiliations:** 1 Medicine and Epidemiology, University of California-Davis, School of Veterinary Medicine, Davis, CA USA; 2 Dean’s Office, University of California-Davis, School of Veterinary Medicine, Davis, CA USA; 3 Surgical and Radiological Sciences and Dean’s Office, University of California-Davis, School of Veterinary Medicine, Davis, CA USA

**Keywords:** Veterinary, Students, Burnout, Maslach

## Abstract

**Background:**

Burnout among veterinary students can result from known stressors in the absence of a support system. The objectives of this study were to evaluate use of the Maslach Burnout Inventory-Educator Survey (MBI-ES) to assess burnout in veterinary students and evaluate the factors that predict the MBI-ES scores.

**Methods:**

The MBI-ES was administered to first (Class of 2016) and second year (Class of 2015) veterinary medical students during the 2012-2013 academic year in the fall and spring semesters. Factor analysis and test reliability for the survey were determined. Mean scores for the subscales determining burnout namely emotional exhaustion (EE), depersonalization (DP) and lack of personal accomplishment (PA) were calculated for both classes in the 2 semesters. Multiple regression analysis was performed to evaluate other factors that predict the MBI-ES scores.

**Results:**

A non-probability sampling method was implemented consisting of a voluntary sample of 170 and 123 students in the fall and spring semesters, respectively. Scores for EE, DP and PA were not different between the 2 classes within the same semester. Mean ± SD scores for EE, DP and PA for the fall semester were 22.9 ± 9.6, 5.0 ± 4.8 and 32.3 ± 6.7, respectively. Mean ± SD scores for EE, DP and PA the spring semester were 27.8 ± 10.7, 6.5 ± 6.1and 31.7 ± 6.8, respectively. The EE score was higher in spring compared to fall while DP and PA scores were not different between the 2 semesters. Living arrangements specifically as to whether or not a student lived with another veterinary medical students was the only variable significantly associated with the MBI-ES scores. Students in this study had moderate levels of burnout based on the MBI-ES scores.

**Conclusions:**

The MBI-ES was an acceptable instrument for assessing burnout in veterinary medical students. The EE scores were higher in the spring semester as compared to the fall semester. Thus students in the first and second years of veterinary school under the current curriculum experience the greatest levels of emotional exhaustion during the spring semester. This has administrative implications for the school, when considering the allocation and use of resources for student support systems during each semester.

## Background

The level of stress among veterinary medical students is significant, and is related to the high intense level of training in veterinary medical schools [1]. Reported stressors among veterinary medical students include unsatisfactory family and personal relationships, debt and financial self-insufficiency, excessive workload, lack of time for social and recreational activities [2], chronic sleep deprivation, time demands, experience of constant academic evaluation [1], homesickness, academic concerns, difficulty fitting in with peers and perceived poor physical health [3]. In addition to the stress experienced by veterinary medical students, one study indicated that 32% of first year students experienced clinical levels of depressive symptoms and elevated anxiety levels [4] compared to medical students (14.3%) [5] and the US general population (6.7%) [6]. In addition, preliminary studies indicated that veterinarians have higher rates of death by suicide compared to the general population in several countries including the US, the UK, Scotland, Wales and Australia [7].

Burnout is considered the result of being stressed and not having a sufficient support system [8]. Burnout is also defined as a syndrome characterized by emotional exhaustion, depersonalization (negative, cynical attitudes and impersonal feelings towards others) and reduced personal accomplishment (the tendency to evaluate oneself negatively particularly with regard’s to one’s work) [9]. The Maslach Burnout Inventory (MBI) [9] is considered a reference instrument for measuring burnout in professional occupations including health care [10]-[12], social services [13],[14], mental health [15], criminal justice [16] and education [17]. Studies assessing burnout in practicing veterinarians have been reported [18]-[22]. The MBI has been used to assess burnout in medical students [23]-[27] but the instrument has not been utilized in veterinary medical students. To authors’ knowledge only a single study [28] reported assessment of burnout in veterinary medical students in the US using burnout scores developed by Pines and Aronson [29].

### Current study

The University of California, Davis School of Veterinary Medicine implemented a revised curriculum in 2011 that focused on enhancing students’ ability to integrate learning material across disciplines with the intention of reducing the amount of stress, as measured by burnout, experienced by students during each semester of the new curriculum in order to determine appropriate resource allocation [30]. The first graduating class under the revised curriculum will be the Class of 2015. Prior to the implementation of the revised curriculum, students had a perception of increased workload as a result of the curriculum design, among other factors [31]. Due to excessive workload being identified as a risk for stress [2] and consequently burnout, the objectives of the current study were 2-fold:

1. Evaluate the use of Maslach Burnout Inventory Educator Survey (MBI-ES) for assessing burnout in veterinary medical students and evaluate the factors that best predict MBI-ES scores.
2. Compare the MBI-ES scores between veterinary medical students during the fall and spring semesters to evaluate the level of stress experienced by students during each semester.

Currently, evidence exists supporting that emotional stress is prevalent in veterinary medical students. Thus investigation of the optimal tool to assess the level of burnout will assist in the determination and allocation of student support resources.

## Methods

### MBI-ES questionnaire administration

The study research was approved by the University of California, Davis Institutional Review Board. The survey was delivered using the web-based application, SurveyMonkey. The link to the survey was placed on VIPER (Veterinary Information Portal and Educational Resources) which is a locally developed and administered authenticated collection of web applications that students at University of California, Davis, School of Veterinary Medicine access and use throughout their veterinary medical education. Students were sent an electronic invitation via email informing them of the survey, its purpose and the dates that the survey were available, should they wish to participate. While student responses to the survey were not anonymous as they were tracked through VIPER, all identifiable student information was stripped upon analysis by the researchers and kept confidential.

The MBI-ES is a 22-item instrument [9]. The instrument consists of three subscales to evaluate each domain of burnout, namely emotional exhaustion (EE), depersonalization (DP) and low personal accomplishment (PA). In this study, emotional exhaustion assessed feelings of being emotionally overextended and exhausted by schoolwork. The depersonalization subscale measured an impersonal response towards other students. The personal accomplishment subscale assessed feelings of competence and successful achievement in a student’s schoolwork. Sampling consisted of a non-probability sampling method via the use of a voluntary sample, which was administered to 2 classes (Classes of 2015 and 2016) during 2 consecutive semesters. To account for potential differences in experience of feelings of burnout due to different workloads during different semesters, the survey was administered to both classes during their first semester (fall of 2011 for Class of 2015 and fall of 2012 for Class of 2016). The survey was re-administered to the Class of 2015 during their 4^th^ semester (spring 2013) and to the Class of 2016 during their 2^nd^ semester (spring 2013). The survey was open for a period of 2 weeks during each administration. This survey administration schedule allowed for the veterinary school to assess the level of burnout for 1^st^, 2^nd^ and 4^th^ semesters of the new curriculum. The survey was not administered to the Class of 2015 during their 3^rd^ semester (fall 2012) as the students were invited to complete other surveys requirements in the revised curriculum and it was likely that the additional survey would not have a high response rate to the voluntary sample of the methodology.

### Data analysis

All analyses were performed using commercial statistical software, SAS Version 9.3. Descriptive summary statistics were determined, including the number of students who completed the survey.

### MBI-ES score calculation

Initially, the MBI-ES mean scores for EE, DP and PA were compared between the 2 classes for each semester to check if they differed using a *t*-test for independent groups following confirmation that the scores were normally distributed using the Shapiro-Wilk test. There were no differences in the scores (*P* >0.05) between the 2 classes hence data results from both classes were combined for each semester for subsequent analysis. The MBI-ES scores were calculated as means ± standard deviation (SD) as previously performed in other studies [9]. Comparison between the scores for EE, DP and PA for the fall and spring semester were compared using a *t*-test or Mann-Whitney test.

### Test reliability of the MBI-ES using Cronbach’s alpha

Cronbach’s alpha was determined to assess the reliability of the MBI-ES in the group of students. Reliability scores of ≥0.5 were considered acceptable [32].

### Factor analysis of the MBI-ES

Factor analysis was performed for two reasons. First, it was performed as a data reduction method for subsequent analysis, justify the use of summated scores in the 3 subscales (EE, DP and PA) and evaluate the correlation (loading) between the 22-items in the MBI-ES and EE, DP or PA subscales. Data reduction resulted in the 3 subscales namely EE, DP and PA. Secondly factor analysis was performed to provide validity evidence for the structure of the MBI-ES used in this study. Items with loading of greater than 0.40 were considered significant [9]. In order to account for potential cross-loadings between the EE, DP or PA subscales, oblique rotation of the matrix in the factor analysis was performed [33]. Rotation of the matrix in factor analysis assumed that the 3 subscales were not independent.

### Multiple regression analysis

In order to evaluate the factors that predict the MBI-ES scores, a multiple regression analysis was performed. The dependent variable was the MBI-ES scores. Independent variables considered in the survey included the student’s gender, age of the student at completing the survey, marital status and the living arrangements (whether the student lived with a spouse/partner, another veterinary student, non-veterinary student or lived alone). A stepwise multiple regression was performed with variables with the lowest value of *P* to enter the model added until no variable had a value to enter of *P* <0.1. At each step all variables were evaluated to determine whether they remained significant after addition of any new variables. Initial inclusion of the variable into model was set at *P* <0.1 but a *P* value <0.05 was considered for final comparison.

## Results

One hundred and seventy and 123 students completed the survey in the fall and spring semesters, respectively from both classes. Response rate for the survey was 52.7%. Test reliability coefficients (Cronbach alpha) were 0.60 for EE, 0.52 for DP and 0.77 for PA. Minimum acceptable value for reliability coefficient is 0.5 [32], thus the coefficients indicated acceptable reliability.

Factor analysis results of the 22-items of the MBI-ES are summarized in Table 1. There were similarities and significant differences in the item loading compared to previous studies [9]. Item 1, “I feel emotionally drained from my school”, considered an EE subscale item loaded positively with both EE and DP subscales. Item 6, (“Working with people all day is really a strain for me”) and item 16, (“Working with people directly puts too much stress on me”) considered as EE subscale items loaded positively with DP subscale and negatively with PA. Items 8 (“I feel burned out from school”), 13 (“I feel frustrated by school”) and 20 (“I feel like I’m at the end of my rope”) that are considered EE subscale items loaded positively with both EE and DP subscales. Item 10 (“I’ve become more callous toward people since I started school”) and 11 (“I worry that school is hardening me emotionally”), which are considered DP items loaded positively with both DP and EE subscales. Item 12 (“I feel energetic”), an item considered to be a PA subscale item loaded positively with the PA subscale and negatively with EE subscale. Item 19 (“I have accomplished many worthwhile things in school”), a PA subscale item loaded positively with PA subscale and negatively with DP. Finally, item 21 (“In school, I deal with emotional problems very calmly”), an item considered to be a PA item did not load significantly with PA, DP or EE subscales. Correlations among the 3 subscales are summarized in Table 2. Personal accomplishment subscale was negatively correlated with both EE and DP subscales while the EE and DP subscales were positively correlated.

**Table 1:** **Summary of the 22**-**item factor analysis loadings for the MBI**-**ES in veterinary medical students (N** = **170). **

This table has been removed because the authors had not obtained permission to reproduce the Maslach Burnout Inventory Educator Survey (MBI-ES). The results presented in this table are available by contacting the authors.

**Table 2:** **Intercorrelations between EE**, **DP and PA subscales in veterinary medical students (N** = **170). **

	Emotional exhaustion	Depersonalization
Depersonalization	0.63	
Personal accomplishment	-0.2	-0.23

The MBI-ES scores for the students are summarized in Table 3. Scores for EE were higher (*P* = 0.0002) in the spring semester compared to the fall semester. Scores for DP (*P* = 0.0526) and PA (*P* = 0.0898) were not different between the fall and spring semesters. Burnout is reported from low to moderate to high degree of experienced feelings [9]. While higher mean scores for EE and DP scale correspond to higher degrees of burnout, low PA scores correspond to higher degrees of burnout [9]. A high degree of burnout is indicated by high scores on EE and DP and low scores on PA [9]. A moderate degree of burnout is reflected by moderate scores on EE, DP and PA while a low degree of burnout is indicated by low scores on EE and DP and high scores on PA [9]. The students in this study had a moderate EE score, moderate DP score and low PA scores. Thus, the group of students in this study was considered to have a moderate degree of experienced feelings of burnout.

**Table 3:** **Mean** ± **SD for the MBI**-**ES scores in veterinary medical students for the fall and spring semesters**

	Emotional exhaustion (EE)	Depersonalization (DP)	Personal accomplishment (PA)
Fall semester (N =170)	22.9 ± 9.6^a^	5.0 ± 4.8^b^	32.3 ± 6.7^c^
Spring semester (N =123)	27.8 ± 10.7^b^	6.5 ± 6.1^b^	31.0 ± 6.7^c^
All students (fall and spring)	25.0 ± 10.4	5.6 ± 5.4	31.7 ± 6.8

Means with similar superscripts (a, b or c) for the same MBI-ES subscale are not different (P >0.05) when comparing EE, DP and PA scores between fall and spring semesters. Reported scores for the medical professions for low range of experienced burnout were ≤18 for EE, ≤5 for DP and ≥40 for PA. Reported scores indicating moderate burnout in the medical professions were 19-26 for EE, 6-9 for DP and 34-39 while scores indicating high degree of burnout were ≥27 for EE, ≥10 for DP and ≤33 for PA [9].

Living arrangements, specifically whether a student lived with another veterinary medical students or not was the only independent variable significantly associated with the MBI-ES scores in this group of students based on the results of the multiple regression analysis (Table 4). Veterinary students living with another student were more likely to have scores indicating low degree of burnout.

**Table 4:** **Results of the multiple regression analysis that predicted MBI**-**ES scores in veterinary medical students**

Predictor	Parameter estimate (95% confidence limits)	SEM	***P-value***
Intercept	41.823 (4.0236, 79.609)	19.116	0.03
Gender	-4.417 (-10.891, 2.596)	3.411	0.226
Age	2.565 (-0.463, 5.593)	1.532	0.096
Dependents	5.2 (-11.353, 21.754)	8.374	0.536
Marital status	1.398 (-6.139, 8.934)	3.813	0.715
Living arrangements			
1. Living with a spouse or partner	1.305 (-6.950, 9.60)	0.31	0.755
2. Living with another veterinary student	7.20 (0.673, 13.728)	2.18	0.031
3. Living with a non-veterinary student	0.799 (-5.502, 7.101)	0.25	0.802
4. Living alone	0.905 (-7.764, 9.574)	0.21	0.837

Goodness of fit for the multiple regression analysis was R^2^ = 0.39.

## Discussion

The main findings in this study was that the MBI-ES was an acceptable instrument to assess burnout in veterinary medical students based on the reliability coefficients for the EE, DP and PA scores and loading of the MBS-ES’s 22 items. The students evaluated in this study had a moderate experience of feelings of burnout. Scores for EE were higher in the spring semester compared to the fall semester. This observation was most likely due to the increased workload in spring due increase in the volume of the learning material because classes commence in fall of each year. Thus, students in the first and second years of veterinary school under the current curriculum experienced the greatest levels of emotional exhaustion during the spring semester. This has administrative implications for the school, when considering the allocation and use of resources for student support systems during each semester. Living with another veterinary student significantly predicted MBI-ES scores suggesting that veterinary students are more likely to be part of the support system to their peers to reduce stress and consequently reducing feelings of burnout. Previous studies indicated that in a 4 year-curriculum, first and fourth year students under 24 years of age, single females in their second year and married females in the fourth years had significantly higher scores for feelings of burnout [28]. It is important to note that previous studies demonstrated test-retest reliability coefficients correlations for the subscales and the test-retest duration ranged from 2 weeks to 1 year [9],[34]-[36]. Thus based on the results of this study and previous studies, we recommended having all students (first to fourth year students) complete the MBS-ES twice a year (fall and spring).

The loading of the MBI-ES, 22 items were similar to previous studies in some aspects [9] but differed significantly in the loading of several items (Table 1). Items 1, 8, 13 and 20 loaded significantly with EE and DP. Emotional exhaustion and DP have been reported to be highly and positively correlated in previous studies [37] and this is consistent in this current study (Table 2). Therefore the loading of the items with both EE and DP could be as a result of the correlation. Furthermore, previous studies indicated that EE and DP were also highly correlated with psychological and physiological strain [37]. There is evidence that veterinary medical students experience psychological and physiological changes during training [3],[38]. Items 6 and 16 which are considered EE items [9] only loaded significantly with DP while other studies reported cross loading by item 16 with both EE and DP [39]. One potential reason for Item 6 (‘Working with people all day is really a strain for me”) and 16 (‘Working with people directly puts too much stress on me”) lacking correlation with EE as expected, could be that the students in this study were in the first or second year of the curriculum and had minimum contact with the school’s employees but interacted mostly with their classmates. However both items 6 and 16 items unexpectedly loaded significantly and negatively with PA and this could be related to how the components of burnout develop. Studies by van Dierendonck and others [40] indicated that PA and DP determine EE, in contrast to studies by Maslach [41], which focused on EE. Thus the studies by van Dierendonck and others [40] suggested that focusing on early signs of EE was not recommended because when the signs of EE appeared, the burnout process was already underway. The mentioned reason may potentially explain why item 6 and 16 loaded significantly with DP and PA and not with EE. Positive correlation between EE and DP (Table 2) most likely explains the cross-loadings of item 10 and 11 with both EE and DP. The results of this study were consistent with previous studies [39] that indicated cross loading of item 12 with EE and PA. In contrast, to previous studies [9], item 19 loaded negatively with DP and positively with PA while no loading with any of the subscales was observed with item 21. Reasons for this difference are unclear.

Although burnout may be inevitable, prevention of burnout requires awareness about the signs of feelings of burnout by the students. Thus, classes for students that cover awareness about burnout and how to manage stress should be emphasized [18]. Previous longitudinal studies involving employees in different fields recommended prevention of burnout by increasing employees’ competence in their job related skills rather than teaching them how to cope with emotional exhaustion [40]. The low PA scores for this group of students indicated diminished personal accomplishment and low PA scores have been identified as a key factor in the development of burnout [42]. In order to increase the sense of personal accomplishment and counteract burnout, specific training programs were recommended for employees in various fields [43]. These training programs included role-playing to provide success experiences (enactive mastery), models of performances (vicarious experiences), coaching and encouragement (verbal persuasion) [43]. Of these recommendations, coaching and encouragement are two aspects that can be incorporated into the stress management classes in the veterinary curriculum. During the implementation of the new curriculum at UC Davis in 2011, such classes were incorporated and integrated into the curriculum. Further studies are required to provide evidence if these classes improve the ability by the students to deal with stress thereby preventing burnout.

It is important to note the limitations of this study. Only 2 classes of the 4-year curriculum were considered in this study due to the technical administration of the survey. Thus the validity of this study maybe limited to these 2 classes and the results of the study may have weak external validity, when other veterinary schools are considered. The survey was voluntary and not random, thus introducing selection bias. Thus students who have experienced feelings of burnout were less motivated to complete the survey or more likely to complete the survey because the topic was relevant to them.

## Conclusions

The MBI-ES can be used as a tool to assess burnout in veterinary medical students. It is practical to use the survey longitudinally (twice a year) in students in various classes of the 4-year curriculum to assess trends and correlations between EE, DP and PA subscales over time. The veterinary medical students in this study had a moderate range of experience feelings of burnout. In order to reduce burnout, school administrators should consider incorporation of classes that make students aware of signs of burnout and help them deal with various stressors during their training. Large numbers of students need to be considered in future studies including involvement of several veterinary schools, if possible, to assess similarities in results particularly loading of the 22-items with the EE, DP and PA subscales and other independent factors predicting the MBI-ES scores.

## Authors’ information

Munashe Chigerwe BVSc, MPH, PhD, DACVIM, is Associate Professor in the Medicine and Epidemiology Department, University of California-Davis, One Shields Avenue, Davis CA 95618 USA. E-mail: mchigerwe@ucdavis.edu. He is a faculty clinician in the Livestock Medicine and Surgery service of the Teaching Hospital and his research interests include neonatal immunity in dairy calves. Karen A. Boudreaux PhD, is Educational Specialist in the Academic Programs Department in the Dean’s Office, University of California-Davis, One Shields Avenue, Davis CA 95618 USA. Jan E. Ilkiw BVSc, PhD, DECVA, is Professor in the Surgical and Radiological Sciences and Associate Dean for Academic Programs, University of California-Davis, One Shields Avenue, Davis CA 95618 USA. She is a companion-animal anesthesiologist and her research interests include pharmacology of injectable anesthetics.

## Abbreviations

MBI-ES: Maslach Burnout Inventory-Educator Survey
EE: Emotional exhaustion
DP: Depersonalization
PA: Personal accomplishment
