# Correction to: Assessment of burnout in veterinary medical students using the Maslach burnout inventory-educational survey: a survey during two semesters

**DOI:** 10.1186/s12909-020-1941-z

**Published:** 2020-01-27

**Authors:** Munashe Chigerwe, Karen A. Boudreaux, Jan E. Ilkiw

**Affiliations:** 1 Medicine and Epidemiology, University of California-Davis, School of Veterinary Medicine, Davis, CA USA; 2 Dean’s Office, University of California-Davis, School of Veterinary Medicine, Davis, CA USA; 3 Surgical and Radiological Sciences and Dean’s Office, University of California-Davis, School of Veterinary Medicine, Davis, CA USA

**Correction to: BMC Med Educ**

**http://www.biomedcentral.com/1472-6920/14/255**

Following publication of the original article [1], we’ve been notified by an author that they have published their manuscript without seeking permission for the survey that was included in one of their tables (Table 1).

Table 1 to be obscure. The legend to be replaced by the following text:

Summary of the 22-item factor analysis loadings for the MBI-ES in veterinary medical students (*N* = 170). This table been removed because the authors have not obtained permission to reproduce the Maslach Burnout Inventory Educator Survey (MBI-ES). The results presented in this table are available by contacting the authors.
