# Correction to: Assessment of burnout in veterinary medical students using the Maslach Burnout Inventory-Educational Survey: a survey during two semesters

**DOI:** 10.1186/s12909-020-02369-x

**Published:** 2020-11-20

**Authors:** Munashe Chigerwe, Karen A. Boudreaux, Jan E. Ilkiw

**Affiliations:** 1 Medicine and Epidemiology, University of California-Davis, School of Veterinary Medicine, Davis, CA USA; 2 Dean’s Office, University of California-Davis, School of Veterinary Medicine, Davis, CA USA; 3 Surgical and Radiological Sciences and Dean’s Office, University of California-Davis, School of Veterinary Medicine, Davis, CA USA

**Correction to: BMC Med Educ 14, 255 (2014)**

**https://doi.org/10.1186/s12909-014-0255-4**

Following publication of the original article [1], the authors became aware that they had not obtained permission to reproduce the Maslach Burnout Inventory Educator Survey (MBI-ES) from the current copyright holder MindGarden (www.mindgarden.com). The table in this Correction replaces Table 1 from the original article.

This Correction supersedes the previous Correction [2] to this article.

**Table 1:** Summary of the 22-item factor analysis loadings for the MBI-ES in veterinary medical students (*N* = 170)

	Emotional exhaustion	Depersonalization	Personal accomplishment
Emotional exhaustion			
Item 1	0.83	0.41	− 0.24
Item 2	0.82	0.33	− 0.18
Item 3	0.79	0.28	− 0.04
Item 6	0.37	0.65	− 0.42
Item 8	0.9	0.49	− 0.17
Item 13	0.74	0.42	− 0.25
Item 14	0.64	0.37	0.04
Item 16	0.36	0.7	− 0.45
Item 20	0.75	0.4	− 0.12
Depersonalization			
Item 5	0.25	0.61	− 0.09
Item 10	0.54	0.79	− 0.27
Item 11	0.56	0.68	− 0.19
Item 15	0.25	0.7	− 0.06
Item 22	0.35	0.51	0.08
Personal accomplishment			
Item 4	0.07	− 0.14	0.61
Item 7	0.27	− 0.2	0.55
Item 9	− 0.11	0.03	0.58
Item 12	− 0.5	− 0.23	0.49
Item 17	− 0.21	− 0.14	0.78
Item 18	− 0.19	− 0.23	0.76
Item 19	− 0.21	− 0.4	0.51
Item 21	− 0.13	− 0.29	0.29

Items with loading of ≥ 0.4 are considered significant
